# Magnitude and predictors of hospital admission, readmission, and length of stay among patients with type 2 diabetes at public hospitals of Eastern Ethiopia: a retrospective cohort study

**DOI:** 10.1186/s12902-021-00744-3

**Published:** 2021-04-19

**Authors:** Lemma Demissie Regassa, Assefa Tola

**Affiliations:** Department of Epidemiology and Biostatistics, College of Health and Medical Sciences, Haramaya University, P. O. Box 135, Dire Dawa, Ethiopia

**Keywords:** Type 2 diabetes, Hospital admission, Length of hospital stay, Readmission, Follow up

## Abstract

**Background:**

Type 2 Diabetes (T2D) represents one of the leading causes for hospital admissions and outpatient visits. Hence, T2D continuously imposes a significant burden to healthcare systems. The aim of this study was to assess predictors of hospital admission, readmission rates, and length of hospital stay among T2D patients in government hospitals of Eastern Ethiopia from 2013 to 2017.

**Methods:**

This study utilized retrospective data from a cohort of T2D patients following their treatment in government hospitals in Harari regional state of Ethiopia. Predictor of hospital admission was determined using parametric survival analysis methods. The readmission rate and length of hospital stay were determined by Poisson regression and mixed effect Poisson regression, respectively. All association were performed at 95% confidence level. Significance of association with determinants was reported using the hazard rate for hospital admission, and the incidence rate for readmission and length of hospital stay. Optimal model for each outcome was selected by using information criteria after fitness was checked.

**Results:**

The hospital admission rate for T2D patients was 9.85 (95%CI: 8.32, 11.66) per 1000-person-year observation. Alcohol drinking, inactive lifestyle, being a rural resident, history of comorbidities, and experiencing chronic diabetes complications were predictors of hospital admission. Seventy-one (52.2%) of the admitted patients had a history of readmission. Readmission rate was increased by being female, duration of disease, inactive lifestyle, having BMI greater than 29.9 kg/m^2^, and higher blood glucose. The median time of hospital stay for admitted patients was 18 (IQR:7). The length of hospital stay was longer among females, patients with the history of insulin administration, and higher blood glucose.

**Conclusion:**

Multiple and complex factors were contributing for high diabetes admission and readmission rates as well as for longer in-hospital duration among T2D patients in Harari regional state. Socio-demographic characteristics (sex, place of residence), behavioral factors (alcohol intake, lifestyle), and medical conditions (longer duration of disease, comorbidities, chronic diabetes complications, higher blood glucose level, and treatment modality) were significant determinants of hospital admission, readmission and longer hospital stay among T2D patients.

## Background

Type 2 diabetes (T2D) is the most common type of diabetes, which is characterized by insulin resistance or scarce insulin activity due to a progressive loss of b-cell insulin secretion [1–4]. Patients with diabetes usually experience comorbidities and complications more frequently than other patients [5]. Therefore, T2D patients have considerably higher rates of admission and longer in-hospital stay than the general population, which imposes a significant burden on healthcare systems [3, 6–10].

Over a million diabetes were admitted to a hospital in England and Wales in 2017, during which one in six hospital beds were occupied by patients with diabetes and by 2030 it is predicted this will rise to one in four [8, 11].

The hospitalization of people with diabetes is commonly precipitated by various health-related conditions. The principal reasons for admission among diabetes are related to acute and chronic complications of diabetes [7, 10–16]. Circulatory disorders such as congestive heart failure, coronary atherosclerosis, and acute myocardial infarction, were the main reasons for hospital admission [5]. Predictors of hospital admission among DM are older age, being less educated, low socioeconomic status, high alcohol use, longer diabetes mellitus (DM) duration, and disease severity [9, 14, 17].

Studies showed that high and middle income countries reported diabetes patients stayed in the hospital for an additional 1–6 days than other patients [6, 8, 11, 13, 18] and longer in low income countries [7, 12, 19]. In Ethiopia, the median duration of hospital stay among DM patients was 9 days (interquartile range of 14 days) ranged from 1 to 88 days [15]. Poor health care service processes, occurrence of co–morbidity, and high rate of complications in diabetes are responsible for these excess lengths of stay in DM patients [5, 12, 13, 15, 20]. Beside these figures there is little information about the burden of hospitalization of diabetic patients in Eastern Ethiopia. In addition, it is valuable to identify the main causes of hospital admission and duration of hospitalization among DM. Therefore, this study aimed to assess the magnitude and predictors of hospital admission, readmission rates, and length of hospital stay among type 2 diabetes in public hospitals of Harar, Eastern Ethiopia.

## Methods

### Study area, design and period

A hospital based retrospective follow-up study was conducted among T2D patients at public Hospitals of Harar, Eastern Ethiopia. Harari Region is one of the 10 regional states of Ethiopia found at 525 km east of Addis Ababa and 55 km southeast of Dire Dawa. The majority of the population are urban dwellers. In the region, there are three government hospitals with established treatment of diabetes under the chronic follow-up clinic. These hospitals are Hiwot Fana Specialized University Hospital (HFSUH), Jugal General Hospital (JGH), and Harar Federal Police Hospital (HFPH). This study used the follow-up data of T2D at those three government hospitals from January 1, 2013, to December 31, 2017.

### Subjects and eligibility criteria

**Fig. 1:**
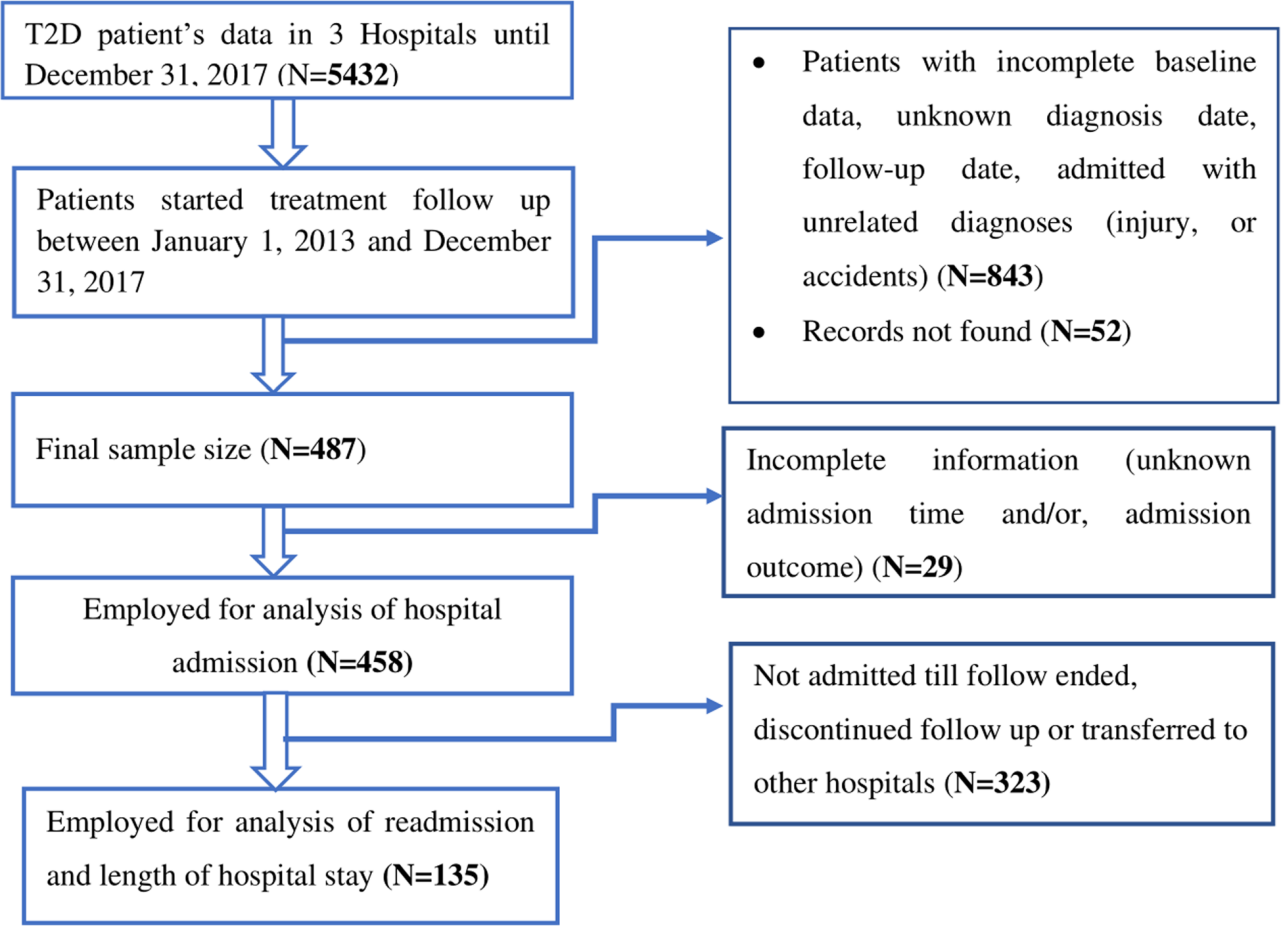
Schematic presentation of sampling and patient selection from the patients on follow-up among Government Hospitals in Harari region, Eastern Ethiopia, from January 1, 2013, to December 31, 2017. Source (HMIS department of each Hospital). Legends: N; number of patients, T2D; Type 2 Diabetes

T2D patients who started follow up from January 1, 2013, to December 31, 2017, was considered as the study population. Patients diagnosed as T2D on admission, patients with unknown admission status, or with incomplete records (baseline data, date of diagnosis, date of follow-up, date of the event, or admitted with unrelated diagnoses (injury, or accidents)) were excluded. Additionally, patients with end-stage kidney disease, on transplantation in other hospitals, patients with known advanced cancer were excluded to avoid estimation bias (Fig. 1).

### Sample size determination and sampling method

The sample size was calculated using hospital admission as main outcome of this study. PASS 2020 software was used to calculate sample size. We employed the assumption of the log-rank test [21], and cox regression [22] to calculate the sample. Accordingly, the minimum number to get the adequate event, the probability of event and sample size are calculated by the following formula: [22, 23]

$$ N=\frac{E\left({\alpha}_1B,\psi \right)}{p_E\left({s}_t,{L}_t,R,T\right)} $$

Where: E() is the number of events required to be observed in a study, and pE() is the probability of observing an event in a study. Hence, by assuming the 5% threshold probability for rejecting the null hypothesis (Type I error rate), a 20% probability of failing to reject the null hypothesis under the alternative hypothesis (Type II error rate), a 10% censoring probability, and 18.8 per 100 people per year proportion of subjects that are T2D [3]. The calculated sample size required to detect 228 minimum number of event was 314. Considering the 15% incomplete chart in those hospitals, the final minimum sample size was 361. However, we included all available data of 487 eligible records of the T2D patients. The records of the patients were included from each hospital (HFSUH, JGH, and HFPH) using Medical Record identifiers.

### Data collection methods

We used secondary data, including patient intake forms, follow up records and DM registration books, as well as electronic information databases, recorded routinely by the hospitals for the following monitoring and evaluation purposes. Data were collected using checklists by health officers and nurses from those three hospitals.

### Data quality control

The overall activity was controlled by the principal investigators of the study. The data collection team was trained on the data collection process for a couple of days. Supervisors checked the ID of the patient with the registry code whether clinically matched (diabetic patient) or not. In addition to these, the supervisor conducted continuous supervision, completeness check, accuracy, and consistency of data collection on each day of the data collection period. Data were cleaned and filled using Epidata version 3.1 statistical software. All completed data were examined for completeness and consistency during data storage and analysis.

### Measurements and variables

Events: The first hospital admission was accounted as an event. Admissions were defined as any unscheduled inpatient admission, emergency department (ED) visit or observation unit stay [24]. Readmission is defined as the admission of T2D patients for more than one time. Time: Was considered as the interval between the time patient started follow up and end of the study period. Initial time was started from the date of follow-up initiation. Duration of disease: refers to the time since the patient was diagnosed as a T2D patient. The duration of the disease was computed from the difference of the event date and date of diagnosis. Duration of follow-up: refers to the time since the T2D patient linked to the diabetes clinic and started follow-up visits. Duration of follow up was computed from the difference last follow-up and date of the first follow-up.

Censoring: an individual was termed as censored if he/she was died or transferred to another follow-up center, or discontinued follow-up, or not admitted to the hospital until the end of this study period.

Combined treatment: The combination of glyburide and metformin is used to treat type 2 diabetes in people whose diabetes cannot be controlled by diet and exercise alone. Baseline serum creatinine (Sc) were classified as Normal if 0.6–1.1 mg/dL in females and 0.8–1.3 mg/dL in males otherwise elevated [25]. BMI: based on WHO classification, BMI was classified as underweight (< 18.5 kg/m^2^), normal (18.5 to < 25 kg/m^2^), overweight (25.0 to < 30 kg/m^2^) and obesity (≥30 kg/m^2^) [26, 27]. Comorbidities: was considered if T2D patients had a previous medical history of at least one or more chronic conditions like hypertension, obesity, dyslipidemia, cardiovascular disease, and/or chronic kidney disease [28]. We classified the number of comorbidities as none, one, two, or more than two by counting the medical history of comorbidities. Chronic complications of diabetes: in this study, chronic diabetes complications were considered if T2D patients had a medical history of at least one chronic microvascular or macrovascular complication related to Diabetic Mellitus during or before hospital admission [29]. Microvascular complications of diabetes are those long-term complications that affect small blood vessels. These typically include retinopathy, nephropathy, and neuropathy [30, 31]. Microvascular complications of diabetes are ischemic heart disease, peripheral vascular disease, and cerebrovascular disease [32, 33].

Lifestyle: in this document the lifestyle was classified using the occupational history and/or daily physical activities [34, 35]. Thus, the patient was termed as active if the patient had a history of reported exercise for at least 30 min per day or working in the field. Otherwise, the patient was termed as inactive.

Baseline variables: sex, residence, occupation, medical history of comorbidities, insulin administration, serum creatinine, and lifestyle were baseline covariates recorded at the first follow up time. Follow up variables: Variables like age, body mass index, fasting blood glucose, treatment type, diabetic complications, smoking status, and disease duration are taken from the most recent (for the event) follow-up record. Blood glucose level: The most recent (to the event) recorded blood glucose level was considered as considered for the analysis.

### Methods of statistical analysis

The collected data were entered into Epi Info v7.0 and analyzed by STATA software version 16.0. Sociodemographic, disease characteristics, and treatment were summarized by proportions. The hospital admission, readmission, and length of hospital stay were summarized by mean, median, and rate. Survival data were described by median time, follow-up time, and Kaplan Meier graphs.

The baseline proportional hazard was assumed constant and Cox proportional hazard assumption was tested for all included variables. As the proportional hazard assumption failed, the appropriate model was selected by inspecting the graphical distribution pattern information criteria (AIC and BIC). Accordingly, the Gompertz parametric model was selected as the best fitted model from Cox regression, time variant Cox regression, exponential regression, Weibull regression, and log-logistic regression models. Hence, the predictors of time to first hospital admission are determined by Gompertz parametric model. Determinants of readmission and length of hospital stay were determined by Poisson mixed effect model (Poisson GLMM) using patient ID as a random effect. The hazard rate was used to report the strength of association for time to hospital admission and its predictors. On the other hand, the relative risk with 95%CI was used to report the association of readmission rate and length of hospital stay’s predictors. All associations were tested at 95% confidence level and *p*-value less than 0.05 were declared as a significant association between variables.

## Results

### Patients characteristics

From the 487 follow up records, 458 (94.05%) records of T2D patients were employed for analysis, and 29 (5.95%) were excluded due to incomplete information regarding the admission records. Of the total, 227 (49.56%) from Hiwot Fana Specialized University Hospital, 124 (27.07%) from Jugal General Hospital, and 107 (23.36%) from Harar Police Hospital. The median age of the patients was 50 years with a range of 30 to 69 years. Most (85.15%) of the patients were younger than 65 years. Three hundred eighty-nine (84.93% were urban residents. Most of the participants were married (85.59%). Hundred seventy-eight (40.36%) were government employees (Table 1).

**Table 1:** Socio-demographic and medical Characteristics of T2D patients in governmental hospitals of Harari regional state of Ethiopia (2013–2017)

Variable	Categories	Frequency (%)	Admitted (AR per 1000)	Median LOS (dy.)
Sex	Female	200 (43.7)	60 (9.5)	14
	Male	258 (56.3)	75 (10.1)	7
Age (yrs.)	Less than 45	184 (40.2)	52 (10.2)	7
	45–65	206 (45.0)	58 (9.2)	14
	above 65	68 (14.8)	25 (11.0)	7
Residence	Urban	389 (84.9)	110 (9.0)	7
	Rural	69 (15.1)	25 (17.2)	9
Occupation	Government employee	178 (40.3)	40 (7.1)	7
	Private job	163 (37.0)	54 (11.8)	7
	Unemployed	100 (22.7)	29 (9.9)	17.5
Smoking	Non smoker	444 (96.9)	127 (9.6)	7
	Current smoker	14 (3.1)	8 (16.1)	21
Alcohol	Not drink alcohol	430 (93.9)	122 (9.5)	7
	Drink alcohol	20 (6.1)	13 (15.5)	7.5
Blood glucose level	80–130 mg/dl	327 (71.4)	95 (9.8)	7
	> 130 mg/dl	131 (28.6)	40 (9.9)	14
Baseline Creatinine	Normal	142 (31.0)	42 (9.9)	7
	Elevated	316 (69.0)	93 (9.9)	7.5
Time between diagnoses and first follow up	< 1 month	154 (86.03)	114 (8.7)	10
	1–3 months	11 (6.15)	9 (10.2)	7
	> 3 months	14 (7.82)	12 (12.6)	7
Microvascular Complications	Yes	388 (84.7)	104 (9.2)	7
	No	70 (15.3)	31 (12.8)	11
Comorbidity	No	284 (62.0)	72 (9.0)	7
	Yes	174 (38.0)	63 (11.0)	10.5
No. of comorbidities (*n* = 174)	One	123 (70.7)	37 (6.9)	7
	Two	42 (24.1)	20 (11.5)	14
	Three or more	9 (5.2)	6 (17.6)	14
Recorded Insulin Administration	No	404 (88.2)	110 (9.2)	7
	Yes	54 (11.8)	25 (14.8)	10.5
Medication	Metformin	231 (50.4)	73 (10.9)	7
	Combined	227 (49.6)	62 (8.9)	7
Total		458	135 (9.85)	7

Note: *AR* Admission rate, *mg/dl* milligram per deciliter

Of the total, 167 (36.46%) lived inactive life and 113 (24.89%) had a family history of diabetes. Only 2.84% were active smokers, and 6.11% had a history of alcoholism. During their lifetime, 72 (15.86%) of the patients had a history of acute complications (diabetic ketoacidosis or Hyperosmolar hyperglycemic state) and 74 (16.16%) had chronic complications (15.28% microvascular complications). Of the total, 174 (37.99%) had hypertension, 61 (13.32%) had a history of obesity, 23 (4.58%) had history of a cardiovascular disease, and 27 (5.38%) had a history of renal disease (Table 1).

### Hospital admission rate and predictors

**Fig. 2:**
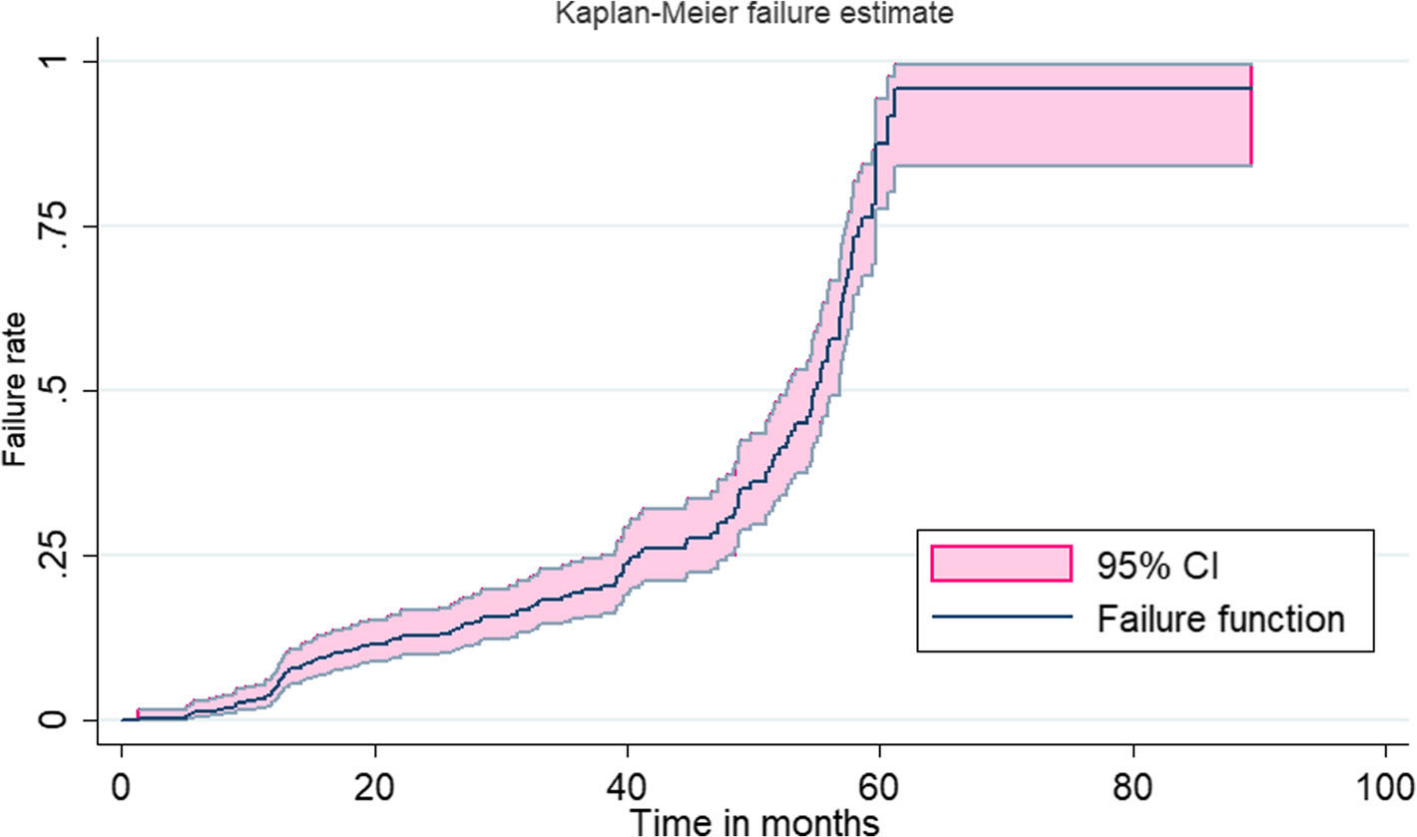
Kaplan Meier Hospital admission rate for type 2 diabetes patients following their treatment at government hospitals of Harari regional state of Ethiopia (2013–2017). Legend: CI; confidence interval

Study subjects were followed for a median time of 54.87 months (IQR: 38.7) after initiation of treatment for a total of 13,703.9 person-years. From the total, 135 (29.48%) had a history of admission with an admission rate of 9.85 (8.32, 11.66) per 1000-person year observation. The cumulative probability of first hospital admission among type 2 DM patients was 0.09 at 15 months, 0.35 at 48 months, and 0.89 at 60 months of follow up period (Fig. 2). Most patients were admitted with hypertension emergency (47%) and acute metabolic complications (either ketoacidosis/ hyperosmolarity) (24.6%). The other reasons for admission were kidney injury (10.5%), infections (pneumonia or severe urinary infection) (9.7%), microvascular complications (6%), and stroke (2.2%). Most (95.6%) of the admitted patients were survived and discharged from the hospital while the left six (4.4%) patients died.

The best-fitted model was a parametric Gompertzian model with proportional hazard assumptions (Table 2). Multivariable Gompertzian proportional hazard model showed alcohol drinking, inactive lifestyle, being a rural resident, presence of a history of comorbidities, and experiencing chronic diabetes complications were predictors of hospital admission (Table 3).

**Table 2:** Results of survival analysis model comparison for predictors of hospital admission rate among T2D patients

Model	df	AIC	BIC
Cox-proportional hazard	12	11,498.93	11,569.71
Time variant cox	19	11,465.18	11,577.25
Exponential	14	3552.52	3635.09
Weibull regression	15	2154.17	2242.64
Log logistic	15	2391.64	2480.11
Gompertzian	15	1781.62	1870.09

*df* degree of freedom, *AIC* Akaike information criteria, *BIC* Bayesian information criteria

**Table 3:** Predictors of hospital admission among T2D patients on follow up in governmental hospitals of Harari regional state of Ethiopia (2013–2017)

Variables	Categories	Crude HR (95%CI)	Adjusted HR (95%CI)
Age	< 45	1	1
	45–65	0.77 (0.53, 1.12)	0.78 (0.50, 1.20)
	above 65	0.94 (0.58, 1.51)	0.63 (0.37, 1.09)
Sex	Male	1	1
	Female	1.63 (1.42, 1.87) *	0.71 (0.46, 0.96) *
Resident	Urban	1	1
	Rural	2.71 (1.75, 4.21) **	3.57 (2.23, 5.72) **
BMI	< 25 kg/m^2^	1	1
	25–29.9 kg/m^2^	1.86 (0.38, 1.97)	1.09 (0.46, 2.55)
	≥30 kg/m^2^	0.82 (0.34, 1.13)	0.92 (0.49, 1.72)
Smoking	No	1	1
	Yes	1.55 (0.76, 3.17)	0.96 (0.43, 2.19)
Alcohol	No	1	1
	Yes	1.87 (1.05, 3.32) *	2.29 (1.19, 4.38) *
Life style	active	1	1
	Inactive	1.35 (0.96, 1.89)	1.88 (1.26, 2.81) **
Comorbidities	No	1	1
	One	0.84 (0.57, 1.25)	0.91 (0.61, 1.37)
	Two	1.50 (0.93, 2.41)	1.40 (0.84, 2.35)
	More than two	3.17 (1.52, 6.65)	4.46 (1.86, 10.71) **
Diabetes complication	No	1	1
	Yes	1.35 (0.90, 2.01)	1.19 (1.06, 1.59) *
Treatment	Metformin only	1	1
	Combination ^a^	0.86 (0.61, 1.21)	1.04 (0.71, 1.54)
Insulin administered	No	1	1
	Yes	0.87 (0.60, 1.26)	1.33 (0.81, 2.19)
Blood glucose level		1.03 (1.01, 1.04)	1.03 (1.01, 1.04) *
Baseline creatinine		1.01 (0.69, 1.44)	0.87 (0.58, 1.30)
1/gamma			0.07 (0.05, 0.08)

*BMI* body mass index, *kg/m2* kilograms per meter square, *mg/dl* milligram per deciliter, *HR* Hazard rate, *CI* confidence interval, *; *p*-value< 0.05 **; *p*-value< 0.001

^a:^ taking both metformin and glibenclamide

The patients consuming alcohol would have 2.29 times (AHR: 2.29; 95%CI: 1.19, 4.38) higher probability of admitted to hospital than those who did not consume alcohol. Compared with patients who live an active lifestyle, the probability of hospital admission was higher by 88% (AHR: 1.88; 95%CI: 1.26, 2.81) for patients who live inactive lives. The hazard of hospital admission among rural residents was more than three (AHR: 3.57; 95%CI: 2.23, 5.72) times higher than the urban residents. Having a medical history of more than two comorbidities increased the hazard of hospital admission by more than four (AHR: 4.46; 95%CI: 1.86, 10.71) times compared to patients who did not experience comorbidities. Moreover, the hazard of hospital admission was increased by 19% (AHR: 1.19; 95%CI: 1.06, 1.59) among individuals with a history of chronic complications compared to those without chronic complications. The hazard of admission was increased by 3% (AHR:1.03; 95%CI: 1.01, 1.04) per 10 mg/dL increment in fasting blood glucose level (Table 3).

### Reasons and predictors of readmission

**Fig. 3:**
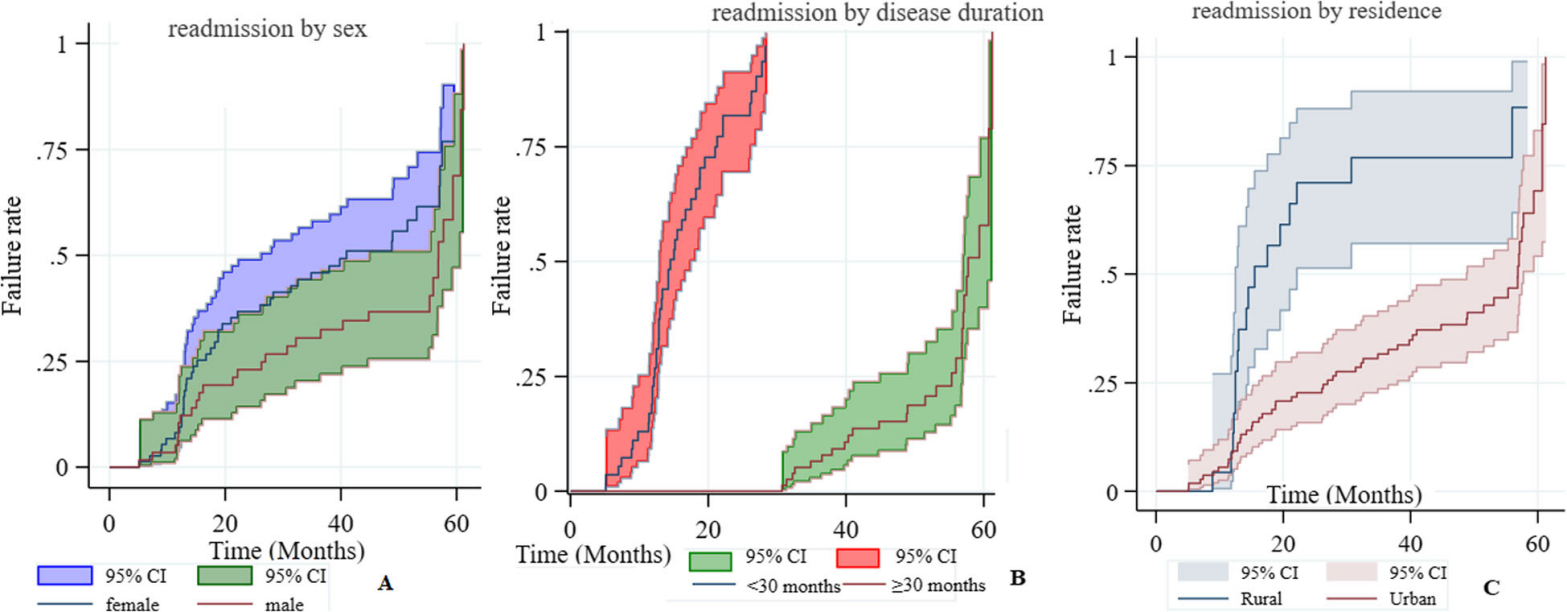
Kaplan Meier of readmission rate of different categories of T2D patients in Government hospitals of Harari regional state of Ethiopia (2013–2017). Legends: panels show failure rate (**a**: readmission rate by sex, **b**: readmission rate by disease duration and **c**: readmission rate by residence), CI; Confidence Interval

Among 135 T2D patients with a history of admission, 71 (52.2%) had readmission within the five years follow up period. After the first admission, the readmission rate was 5.18 (4.11, 6.54) per 1000-person year observation. The readmission rate for female was 12.23 (8.44, 17.71) per 1000-person year observation and 17.41 (12.87, 23.56) among males. The readmission rate was higher among patients from rural areas (4.4 per 1000 for urban Vs 11.7 per 1000 for rural). It is also higher among patients whose duration of the disease is 30 months or more (2.5 per 1000 for less than 30 months Vs 12.5 per 100 30 months and beyond) (Fig. 3). Log rank test also indicated the readmission was higher among patients from rural area (log-rank 15.5, *p* < 0.001), and patients having duration of disease longer than 30 months (log-rank = 145.25, *p* < 0.0001). Log rank analysis showed the difference between male and female was not significant (log-rank = 3.62, *p* = 0.057).

On the other hand, every seven in ten hospital readmissions among T2D patients was attributed to an acute metabolic complication (either ketoacidosis/hyperosmolarity (96.4%) or hypoglycemic coma (3.6%). The other reasons for readmission among T2D patients were cardiovascular complications (18%) and diabetic microvascular complications (12%).

Readmission was determined by the duration of disease, life style, being female, having BMI greater than 24.9 kg/m^2^, and having less than 130 mg/dl average glucose level (Table 4). As the duration of the disease increased by one year, the likelihood of readmission was increased by 14% ((RR: 1.14; 95%CI: 1.05, 1.85). Similarly, being female increases the likelihood of readmission by 26% (RR: 1.26; 95%CI: 1.13, 1.78) compared to male patients. The likelihood of readmission for a currently obese (≥30 kg/m^2^) patient was 2.30 (RR: 2.30; 95%CI: 1.07, 4.96) times higher for those with less than 25 kg/m^2^. The readmission rate was increased by 71% (RR: 1.71; 95%CI: 1.50, 2.02) among patients living inactive lifestyle compared to those living an active life style. In addition to these, the likelihood of readmission was increased by 2% (RR: 0.46; 95%CI: 1.01, 1.04) as fasting blood glucose level increased by 10 mg/dL.

**Table 4:** Predictor readmission to hospital among T2D patients in governmental hospitals of Harari regional state of Ethiopia (2013–2017) (*n* = 135)

Readmission rate	Categories	Crude RR (95%CI)	Adjusted RR (95%CI)
Duration of T2D (year)		1.09 (1.04, 1.15) **	1.14 (1.05, 1.85) **
Smoking status	Non smoke	1	1
	smoker	1.16 (0.82, 1.63)	1.12 (0.51, 2.50)
Alcohol consumption	No	1	1
	Yes	1.06 (0.81, 1.40)	0.83 (0.43, 1.62)
Life style	Active	1	1
	Inactive	1.18 (1.02, 1.35) *	1.71 (1.50, 2.02) *
Sex	Male	1	1
	Female	1.29 (0.93, 1.79)	1.26 (1.13, 1.78) **
BMI	< 25 kg/m^2^	1	1
	25–29.9 kg/m^2^	1.40 (0.52, 3.766)	1.45 (0.53, 3.965)
	≥30 kg/m^2^	1.92 (0.90, 4.13)	2.30 (1.07, 4.96) *
Blood glucose level	70–130 mg/dl	0.45 (0.37, 0.55) **	1.02 (1.01, 1.04) **
Treatment	Metformin	1	1
	Combination ^a^	0.45 (0.31, 0.64)	0.54 (0.37, 0.79) *

*BMI* body mass index, *kg/m2* kilograms per meter square, *mg/dl* milligram per deciliter, *RR* relative risk, *CI* confidence interval, *; *p*-value< 0.05 **; *p*-value< 0.001

^a:^ taking both metformin and glibenclamide

### Length of hospital stay and its predictors

**Fig. 4:**
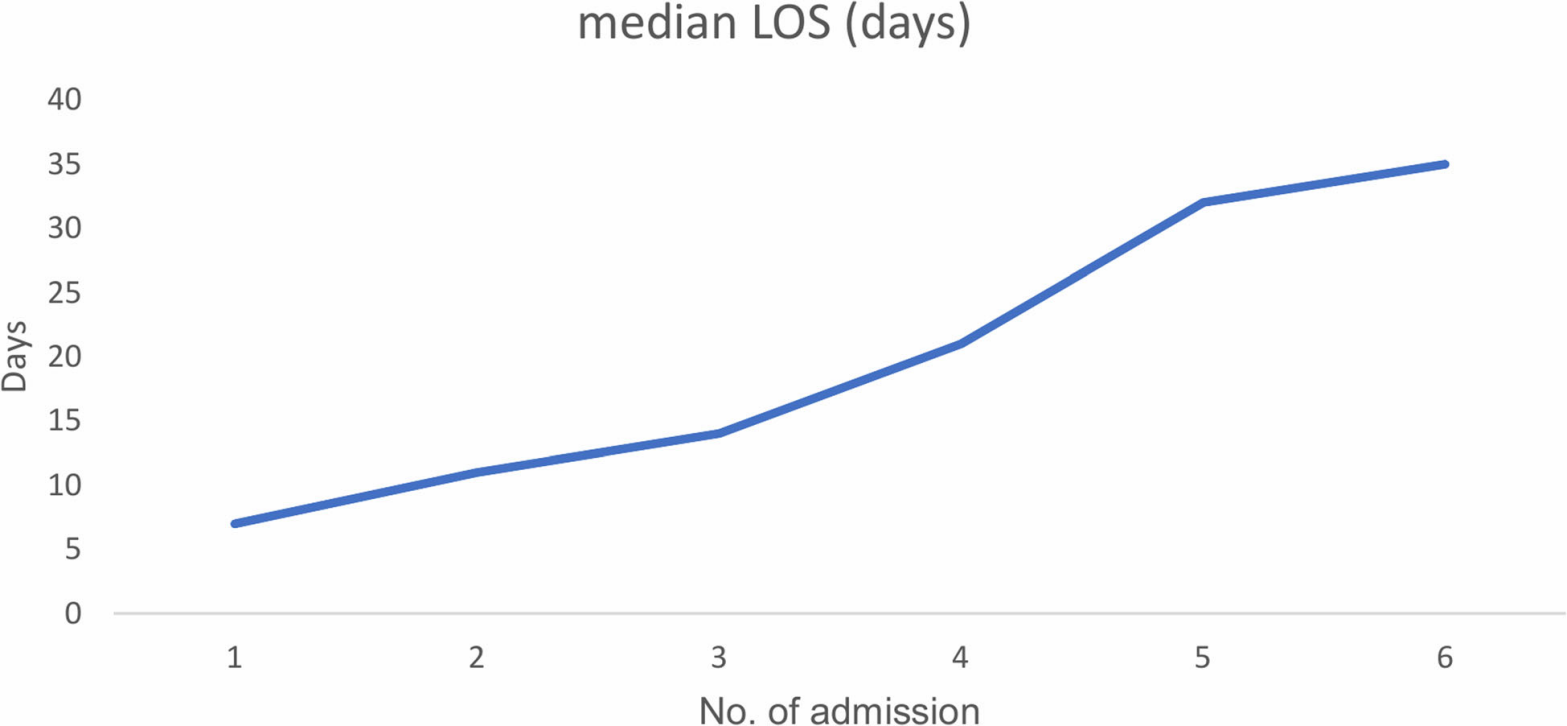
Median length of hospital stay by readmission status of T2D patients among government hospitals of Harari regional state of Ethiopia (2013–2017). Legend: LOS; Length of Hospital Stay

The median time of hospital stay (LOS) for admitted patients was 7 days, ranging from 1 day to 42 days. The median time of LOS among females was higher than males (log rank = 4.4, *p* = 0.035). Median LOS was increasing with the number of readmissions (Fig. 4).

The subject-specific random effects appear significant and patients tend to stay longer in the hospital when they have been hospitalized several times: (variance = 2.03 with standard error 0.22). The length of hospital admission was longer among females, patients with a history of insulin administration, and patients with elevated blood glucose. Being female increases the length of hospital stay by 12% (RR: 1.12; 95%CI: 1.04, 2.52) compared to male patients. Likewise, LOS was increased by 3.41 (RR: 3.41; 95%CI: 1.30, 8.98) times among patients who had a history of insulin administration compared to those who did not take insulin. The risk of LOS was increased by 4% (RR: 1.04; 95%CI: 1.02, 1.07) per 10 mg/dL increase in the fasting blood glucose level. The risk of LOS was decreased by 65% (RR:0.35; 95%CI: 0.14, 0.85) among patients treated with the combination of metformin and glibenclamide compared to patients taking metformin only (Table 5).

**Table 5:** predictors of length of hospital stay among T2D patients at government hospitals of Harari regional state of Ethiopia (2013–2017) (n = 135)

Length of hospital stay	Categories	Median (IQR) LOS (wk.)	Adjusted RR (95%CI)
Sex	Male	0.75 (1.17)	1
	Female	1.09 (1.79)	1.12 (1.04, 2.52) *
Duration of disease (year)		0.92 (1.51)	0.63 (0.49, 1.81)
Smoking	No	0.95 (1.54)	1
	Yes	0.55 (1.02)	0.73 (0.46, 1.16)
Blood glucose level	Per 10 dl/ml	0.92 (1.51)	1.04 (1.02, 1.07) **
Recent BMI	< 25 kg/m2	0.87 (1.42)	1
	25–29.9 kg/m2	1.08 (1.77)	1.46 (0.057, 3.67)
	> 30 kg/m^2^	1.23 (1.75)	2.15 (0.54, 8.55)
Insulin administered	No	0.72 (1.34)	1
	Yes	1.88 (1.89)	3.41 (1.30, 8.98) *
Treatment	Metformin only	1.2 (1.4)	1
	Combination^a^	0.9 (1.32)	0.35 (0.14, 0.85) *
Subject: Variance			2.03 (1.64, 2.52) *

*IQR* Inter quartile range, *LOS* length of hospital, *wk* weeks, *mg/dl* milligram per deciliter, *RR* relative risk, *CI* confidence interval, *kg/m2* kilograms per meter square, *; *p*-value< 0.05 **; *p*-value< 0.001

^a:^ taking both metformin and glibenclamide

## Discussion

This study was designed to assess predictors of hospital admission, readmission, and length of hospital stay among T2D patients on follow up at hospitals of Harari regional state. The five-year incidence of hospital admission was 9.85 per 1000 people per observation time. This result was lower than studies conducted in the United Kingdom (UK) [3], USA [36, 37], and New Zealand [18]. However, our finding was higher than the report from the study conducted in other Sub Saharan Africa like Uganda (4.2% )[38], and Nigeria (10%) [39]. The difference might be due to the difference in health service coverage, health seeking behavior of patients, and admission guidelines/protocols between the countries.

Being a rural resident may increase the probability of hospital admission by more than three times compared to the urban residence. The possible explanation for this finding might be due to the poor diabetic control among T2D from rura l[40, 41]. Similarly, T2D patients from rural areas have low socioeconomic status and opportunity to medical care which make them more prone to hospital admission [42]. The findings of this study, therefore, raise potentially important quality-of-care concerns. Contrary to this finding, the pooled analysis from a study conducted in Poland reported the urban patients have higher probability of hospital admissio n[43]. The difference might be raised from the difference in the study population and the statistical model used. The former study was conducted among patients older than 45 years and compared with patients with malignancy.

Compared to physically active patients, the probability of hospital admission and readmission among patients who live inactive lifestyle were increased by 88 and 71%, respectively. This finding was in line with the report from Australia [44]. The recommended physical activity for T2D is at least 150 min/week of moderate to vigorous aerobic exercise spread out to at least 3 days per week, with no more than two consecutive days between bouts of aerobic activity [45–47]. Otherwise, inactive patients are more prone to insulin resistance, obesity, and comorbidities than diabetes patients living an active life style. As a result, the frequent hospitalization due to those comorbidities and diabetic complications could be higher among patients with inactive life.

Drinking alcohol increases the hazard of hospital admission by 2.3 times higher than patients who did not drink alcohol. Behavioral risk factors in general and alcohol drinking in specific have a significant role in poor diabetic control [6, 48]. Previous studies also indicated that drinkers had the greatest compliance with each self-care behavior, and dehydration induced hypoglycemia [48–50]. Alcohol can cause the accumulation of certain acids in the blood that may result in disturbances in fat metabolism, nerve damage, and eye disease [49].

Having at least one diagnosed chronic diabetic complication increases the probability of hospital admission by about 20% than those who did not have a history of diagnosed diabetic complications. Similarly, having at least three medical histories of comorbidities increased the likelihood of hospital admission by more than four times compared to those who had no diagnosed comorbidities. The finding was comparable with studies conducted in USA [51, 52], UK [3], Australia [9], and Korea [53]. Evidence also indicate that hospital morbidity results from chronic microvascular or microvascular complications [54]. If early screening and preventive activities are implemented, asymptomatic identification of comorbidities and commence treatment are not commenced patients could experience frequent hospitalization [53–56].

As the duration of diabetes increases by one year, the probability of hospital readmission was increased by 14%. The result is similar to the previous reports in Australia [6, 9], and the USA [24]. Diabetes complications and comorbidities are increasing with age and diabetes duration [57–59]. Hence, the frequent hospital admission might correlate with diabetes duration due to the comorbidities and diabetes complications over a long period.

The association between sex and admission, and log rank result for readmission was not significant in univariate but significant in multivariate analyses. The reason of sex be significant when more predictor variables are entered into the model is explained the increment of power and loose of significance. The residual variability decreases in multiple regression since we are comparing the slope to a null distribution that is estimated using the residual variability. Meanwhile the residual degrees of freedom also decrease, so it’s possible that power could be increased in robust model [60].

The hazard of first admission among female T2D was decreased by 29%. However, being female increases the risk of readmission by 26% and LOS by 12% compared to males. This report was contrary to the previous study conducted in Australia [9], USA [36]. A study conducted in India suggested that women had less self-efficiency related to their diabetic self-care and higher chances of bad dietary practices than men [61]. Another study also revealed that females had significantly higher annual rates of symptomatic, glucose-confirmed symptomatic, and severe hypoglycemia than males [62]. All these factors might be increasing the probability of frequent admission and LOS among female patients.

The likelihood of readmission was 2.3 times higher among patients with BMI 30 kg/m^2^ or more compared to patients having BMI less than 25 kg/m^2^. It is consistent with the report of studies from New Zealand [18] and the USA [63]. Previous study also reported that unfavorable diabetes outcome is higher among obese individuals [64].

As the blood glucose level was increased by 10 dl/ml, the risk of admission, readmission, and LOS was slightly increased. This finding was in line with previous studies [65–69]. Uncontrolled blood glucose is the foundation of most diabetic complications and comorbidities among T2D patients [70]. Despite the marker of poor clinical outcome is shown as hyperglycemia [71], it is not proven whether admission hyperglycemia is a risk factor itself, a marker of severity of illness, a marker of poor patient compliance with treatment, or a sign of less rigorous medical care.

The LOS among patients having a history of insulin administration on the admission time was increased by more than three times compared to those who had no history of insulin administration. This finding is in agreement with the report of previous studies [53, 72, 73]. This finding might be related to the side effects of insulin (i.e., hypoglycemia). Hence, cautious monitoring of signs of side effects of insulin administration is important. Insulin might be administered to T2D patients if they have uncontrolled blood glucose levels and DM complications during admission. However, a study conducted among hospitalized elderly patients showed there was no difference in LOS by their insulin administration status [74]. The difference might be due to the difference in the study population as the former study used elderly hospitalized patients.

The combination of metformin and glibenclamide treatment showed a decrement in the number of readmissions and LOS by 46 and 65% respectively. Reports from randomized control trials showed that a combination of treatment is more effective than a single drug in improving glycemic control in type 2 diabetes and reduction of the dosage of each drug [75, 76].

Findings from this study should be interpreted in light of the following limitations. This study neither establish the causality nor representativeness to the whole country as it encountered the problem of small sample size for readmission and LOS, and the limitations of data completeness/accuracy common to most retrospective studies. Furthermore, important determinants like diabetes self-care (diet modification, life style, exercise, etc.) and treatment during hospitalization, organ function tests, and HgA1 were lacked from the chart of the patients. Moreover, we were unable to control for some risk factors, such as laboratory values and hospital acquired complications as these factors were not recorded. In addition to these, we could not get information on medications like anti-hypertensives, fibrates, and statins.

## Conclusions and recommendations

Three out of ten T2D patients had a history of admission and half of those patients with a history of admission were readmitted again within five years. Multiple and complex factors were contributing for high diabetes admission and readmission rates as well as for longer duration in hospital among T2D patients in Harari regional state. Hospital admission was determined by inactive life style, place of residence, presence of a history of comorbidities, and chronic complications while readmission was significantly increased with duration of disease, inactive lifestyle, being female, having a larger BMI, and decreased by having a normal average glucose level. The length of hospital stay was longer for those females, patients with the history of insulin administration, and patients with elevated blood glucose levels. Therefore, health care providers should give due emphasis to diabetes patients with risk factors for admission, readmission, and longer duration in hospital. Effective primary preventive strategies should be implemented at diabetes follow up clinic to reduce the hospitalizations and longer hospital stays among diabetic patients. Health professionals should provide continuous health education for all T2D patients at follow up clinic about the risk factors of hospitalization. All hospitals who provide diabetes care should implement standardized diabetes management guidelines and treatment protocols to avoid unnecessary hospitalization and hospital stay.

## Data Availability

The datasets used and/or analyzed during the current study are available from the corresponding author on reasonable request.

## Abbreviations

AIC: Akaike Information criteria
BIC: Bayesian Information criteria
BMI: Body Mass Index
CI: Confidence interval
DM: Diabetic mellitus
HR: Hazard Rate
IQR: Interquartile Range
LOS: Length of Hospital Stay
RR: Relative risk
T2D: Type 2 Diabetes

## References

[1] American Diabetes Association. Diagnosis and classification of diabetes mellitus. Diabetes Care. 2010;33 Suppl 1(Suppl 1):S62-9. 10.2337/dc10-S062. Erratum in: Diabetes Care. 2010;33(4):e57. 10.2337/dc10-S062 PMC2797383 20042775

[2] American Diabetes Association. 2. Classification and Diagnosis of Diabetes: Standards of Medical Care in Diabetes-2018. Diabetes Care. 2018;41(Suppl 1):S13-S27. 10.2337/dc18-S002. 10.2337/dc18-S002 29222373

[3] Khalid J, Raluy-Callado M, Curtis B, Boye K, Maguire A, Reaney M (2014). Rates and risk of hospitalisation among patients with type 2 diabetes: retrospective cohort study using the Uk general practice research database linked to English hospital episode statistics. Int J Clin Pract.

[4] World Health Organization. Global Report on Diabetes. Geneva: World Health Organization; 2016.

[5] Fraze T, Jiang H, Burgess J (2010). Quality. Hospital stays for patients with diabetes, 2008. Agency Health Care Res.

[6] Comino EJ, Harris MF, Islam MF, Tran DT, Jalaludin B, Jorm L (2015). Impact of diabetes on hospital admission and length of stay among a general population aged 45 year or more: a record linkage study. BMC Health Serv Res.

[7] Faiza H (2019). Reasons for admission of individual with diabetes to the Tripoli medical center in 2015. Diabetes Metab Syndr.

[8] Johnston P (2018). National Diabetes Inpatient Audit: how can inpatient teams make patients feel safer?. Br J Nurs.

[9] Sajjad MA, Holloway KL, de Abreu LL, Mohebbi M, Kotowicz MA, Pedler D (2018). Comparison of incidence, rate and length of all-cause hospital admissions between adults with Normoglycaemia, impaired fasting glucose and diabetes: a retrospective cohort study in Geelong. Australia BMJ open.

[10] Tomlin AM, Tilyard MW, Dovey SM, Dawson AG (2006). Hospital admissions in diabetic and non-diabetic patients: a case-control study. Diabetes Res Clin Pract.

[11] Akbar DH, Al-Gamdi AA (2000). Common causes of admission in diabetics. Saudi Med J.

[12] Ajayi EA, Ajayi AO. Pattern and outcome of diabetic admissions at a federal medical center: a 5-year review. Ann Afr Med. 2009;8(4):271–5. 10.4103/1596-3519.59584. 10.4103/1596-3519.59584 20139552

[13] Allan B, Sampson M (2013). Admissions Avoidance and Diabetes: Guidance for Clinical Commissioning Groups and Clinical Teams.

[14] Begum N, Donald M, Ozolins IZ, Dower J (2011). Hospital admissions, emergency department utilisation and patient activation for self-management among people with diabetes. Diabetes Res Clin Pract.

[15] Kefale AT, Eshetie TC, Gudina EK (2016). Hospitalization pattern and treatment outcome among diabetic patients admitted to a teaching Hospital in Ethiopia: a prospective observational study. J Health Med Nurs.

[16] Unadike B, Essien I, Akpan N, Peters E, Essien O (2013). Profile and outcome of diabetic admissions at the University of Uyo Teaching Hospital, Uyo. Int J Med Med Sci.

[17] Maradzika J, Mzorodzi T, Chikwasha V (2017). Factors associated with hospital admissions among registered diabetes mellitus patients in Guruve and Mazowe District-Mashonaland Central Province, 2013. Int J Health Promot Educ.

[18] Tomlin AM, Dovey SM, Tilyard MW (2008). Risk factors for hospitalization due to diabetes complications. Diabetes Res Clin Pract.

[19] Ncube-Zulu T, Danckwerts M (2014). Comparative hospitalization cost and length of stay between patients with and without diabetes in a large tertiary Hospital in Johannesburg, South Africa. Int J Diabetes Dev Countries.

[20] Cheng S-W, Wang C-Y, Ko Y. Costs and Length of Stay of Hospitalizations Due to Diabetes-Related Complications. J Diab Res. 2019;2019:1–5. 10.1155/2019/2363292. 10.1155/2019/2363292 PMC6754874 31583247

[21] Wu J (2015). Sample size calculation for the one-sample log-rank test. Pharm Stat.

[22] Hsieh FY, Lavori PW (2000). Sample-size calculations for the cox proportional hazards regression model with nonbinary covariates. Control Clin Trials.

[23] Freedman LS (1982). Tables of the number of patients required in clinical trials using the Logrank test. Stat Med.

[24] Ostling S, Wyckoff J, Ciarkowski SL, Pai C-W, Choe HM, Bahl V (2017). The relationship between diabetes mellitus and 30-day readmission rates. Clin Diabetes Endocrinol.

[25] Couchoud C, Pozet N, Labeeuw M, Pouteil-Noble C (1999). Screening early renal failure: cut-off values for serum Creatinine as an Indicator of renal impairment. Kidney Int.

[26] Weir CB, Jan A. BMI Classification Percentile And Cut Off Points. [Updated 2020 Jul 10]. In: StatPearls [Internet]. Treasure Island (FL): StatPearls Publishing; 2021. Available from: https://www.ncbi.nlm.nih.gov/books/NBK541070/. 31082114

[27] WHO Expert Consultation. Appropriate body-mass index for Asian populations and its implications for policy and intervention strategies. Lancet. 2004;363(9403):157–63. 10.1016/S0140-6736(03)15268-3. Erratum in: Lancet. 2004;363(9412):902. 10.1016/S0140-6736(03)15268-3 14726171

[28] American Diabetes Association. Standards of Medical Care in Diabetes-2019 Abridged for Primary Care Providers. Clin Diabetes. 2019;37(1):11–34. 10.2337/cd18-0105. 10.2337/cd18-0105 PMC6336119 30705493

[29] Lotfy M, Adeghate J, Kalasz H, Singh J, Adeghate E (2017). Chronic complications of diabetes mellitus: a mini review. Curr Diabetes Rev.

[30] Faselis C, Katsimardou A, Imprialos K, Deligkaris P, Kallistratos M, Dimitriadis K (2020). Microvascular complications of type 2 diabetes mellitus. Curr Vasc Pharmacol.

[31] Girach A, Vignati L (2006). Diabetic microvascular complications--can the presence of one predict the development of another?. J Diabetes Complicat.

[32] Viigimaa M, Sachinidis A, Toumpourleka M, Koutsampasopoulos K, Alliksoo S, Titma T (2020). Macrovascular complications of type 2 diabetes mellitus. Curr Vasc Pharmacol.

[33] Chawla A, Chawla R, Jaggi S (2016). Microvasular and macrovascular complications in diabetes mellitus: distinct or continuum?. Indian J Endocrinol Metab.

[34] Hu G, Jousilahti P, Barengo NC, Qiao Q, Lakka TA, Tuomilehto J (2005). Physical activity, cardiovascular risk factors, and mortality among Finnish adults with diabetes. Diabetes Care.

[35] Booth FW, Roberts CK, Laye MJ (2012). Lack of exercise is a major cause of chronic diseases. Compr Physiol.

[36] Kim S, Boye KS (2009). Excessive hospitalizations and its associated economic burden among people with diabetes in the United States. Value Health.

[37] Rosenthal MJ, Fajardo M, Gilmore S, Morley JE, Naliboff BD (1998). Hospitalization and mortality of diabetes in older adults. A 3-year prospective study. Diabetes Care.

[38] Bateganya MH, Luie JR, Nambuya AP, Otim MA (2003). Morbidity and mortality among diabetic patients admitted to Mulago hospital, Uganda. Malawi Med J.

[39] Ojobi JE, Dunga J, Ogiator MO, Mbaave P, RNB (2017). Indications and Outcome of Admission of Diabetic Patients into the Medical Wards in a Nigerian Tertiary Hospital-Atwo Year Review. Jos J Med.

[40] Mainous AG, King DE, Garr DR, Pearson WS (2004). Race, rural residence, and control of diabetes and hypertension. Ann Fam Med.

[41] Smith MW, Owens PL, Andrews RM, Steiner CA, Coffey RM, Skinner HG (2016). Differences in severity at admission for heart failure between rural and urban patients: the value of adding laboratory results to administrative data. BMC Health Serv Res.

[42] Stumetz KS, Yi-Frazier JP, Mitrovich C, Briggs EK (2016). Quality of Care in Rural Youth with Type 1 Diabetes: A Cross-Sectional Pilot Assessment. BMJ Open Diabetes Res Care.

[43] Dąbrowski M, Grondecka A (2017). Diabetes as a risk factor of hospitalization in the surgical Ward due to Cancer in the elderly and middle-aged population. Arch Med Sci.

[44] Burke V, Zhao Y, Lee A, Hunter E, Spargo R, Gracey M (2007). Predictors of type 2 diabetes and diabetes-related hospitalisation in an Australian Aboriginal cohort. Diabetes Res Clin Pract.

[45] Colberg SR, Sigal RJ, Fernhall B, Regensteiner JG, Blissmer BJ, Rubin RR (2010). Exercise and type 2 diabetes: the American College of Sports Medicine and the American Diabetes Association: joint position statement. Diabetes Care.

[46] Boulé NG, Haddad E, Kenny GP, Wells GA, Sigal RJ (2001). Effects of exercise on glycemic control and body mass in type 2 diabetes mellitus: a meta-analysis of controlled clinical trials. Jama..

[47] Thomas DE, Elliott EJ, Naughton GA. Exercise for Type 2 Diabetes Mellitus. Cochrane Database Syst Rev. 2006;(3):Cd002968. 10.1002/14651858.CD002968.pub2 PMC8989410 16855995

[48] Ahmed AT, Karter AJ, Liu J (2006). Alcohol consumption is inversely associated with adherence to diabetes self-care Behaviours. Diabet Med.

[49] Emanuele NV, Swade TF, Emanuele MA (1998). Consequences of alcohol use in diabetics. Alcohol Health Res World.

[50] Ekoru K, Doumatey A, Bentley AR, Chen G, Zhou J, Shriner D (2019). Type 2 diabetes complications and comorbidity in sub-Saharan Africans. EClinicalMedicine..

[51] Ahern MM, Hendryx M (2007). Avoidable hospitalizations for diabetes: comorbidity risks. Dis Manag.

[52] Rubin DJ (2018). Correction to: hospital readmission of patients with diabetes. Curr Diab Rep.

[53] Guk M, Choi J (2017). Factors associated with hospitalization among patients with diabetes mellitus. Korean J Adult Nurs.

[54] Jelinek HF, Osman WM, Khandoker AH, Khalaf K, Lee S, Almahmeed W (2017). Clinical Profiles, Comorbidities and Complications of Type 2 Diabetes Mellitus in Patients from United Arab Emirates. BMJ Open Diabetes Res Care.

[55] Chen H, Zhang Y, Wu D, Gong C, Pan Q, Dong X (2016). Comorbidity in adult patients hospitalized with type 2 diabetes in Northeast China: an analysis of hospital discharge data from 2002 to 2013. Biomed Res Int.

[56] Moss SE, Klein R, Klein BE (1999). Risk factors for hospitalization in people with diabetes. Arch Intern Med.

[57] Zoungas S, Woodward M, Li Q, Cooper ME, Hamet P, Harrap S (2014). Impact of age, age at diagnosis and duration of diabetes on the risk of macrovascular and microvascular complications and death in type 2 diabetes. Diabetologia..

[58] Likitmaskul S, Wacharasindhu S, Rawdaree P, Ngarmukos C, Deerochanawong C, Suwanwalaikorn S (2006). Thailand diabetes registry project: type of diabetes, glycemic control and prevalence of microvascular complications in children and adolescents with diabetes. J Med Assoc Thail.

[59] Gedebjerg A, Almdal TP, Berencsi K, Rungby J, Nielsen JS, Witte DR (2018). Prevalence of micro- and macrovascular diabetes complications at time of type 2 diabetes diagnosis and associated clinical characteristics: a cross-sectional baseline study of 6958 patients in the Danish Dd2 cohort. J Diabetes Complicat.

[60] Lo SK, Li IT, Tsou TS, See L (1995). Non-significant in Univariate but significant in multivariate analysis: a discussion with examples. Changgeng Yi Xue Za Zhi.

[61] Shrestha A, Kosalram K, Gopichandran V (2013). Gender difference in Care of Type 2 diabetes. J Nepal Med Assoc.

[62] McGill JB, Vlajnic A, Knutsen PG, Recklein C, Rimler M, Fisher SJ. Effect of gender on treatment outcomes in type 2 diabetes mellitus. Diabetes research and clinical practice. 2013;102(3):167–74. 10.1016/j.diabres.2013.10.001 24183259

[63] Kim S, Boye KS (2009). Obesity and incremental hospital charges among patients with and without diabetes in the United States. Value Health.

[64] Sonmez A, Yumuk V, Haymana C, Demirci I, Barcin C, Kıyıcı S (2019). Impact of obesity on the metabolic control of type 2 diabetes: results of the Turkish Nationwide survey of glycemic and other metabolic parameters of patients with diabetes mellitus (Temd obesity study). Obes Facts.

[65] Umpierrez GE, Isaacs SD, Bazargan N, You X, Thaler LM, Kitabchi AE (2002). Hyperglycemia: an independent marker of in-hospital mortality in patients with undiagnosed diabetes. J Clin Endocrinol Metab.

[66] Golden SH, Peart-Vigilance C, Kao WH, Brancati FL (1999). Perioperative glycemic control and the risk of infectious complications in a cohort of adults with diabetes. Diabetes Care.

[67] Ahmann A (2004). Reduction of hospital costs and length of stay by good control of blood glucose levels. Endocr Pract.

[68] Bhatia V, Wilding GE, Dhindsa G, Bhatia R, Garg RK, Bonner AJ (2004). Association of Poor Glycemic Control with prolonged hospital stay in patients with diabetes admitted with exacerbation of congestive heart failure. Endocr Pract.

[69] Evans NR, Dhatariya KK (2012). Assessing the relationship between admission glucose levels, subsequent length of hospital stay, readmission and mortality. Clin Med (Lond).

[70] Garg R, Bhutani H, Alyea E, Pendergrass M (2007). Hyperglycemia and length of stay in patients hospitalized for bone marrow transplantation. Diabetes Care.

[71] Gebreegziabher Y, McCullough PA, Bubb C, Loney-Hutchinson L, Makaryus JN, Anand N (2008). Admission hyperglycemia and length of hospital stay in patients with diabetes and heart failure: a prospective cohort study. Congest Heart Fail.

[72] Soh JGS, Wong WP, Mukhopadhyay A, Quek SC, Tai BC (2020). Predictors of 30-Day Unplanned Hospital Readmission among Adult Patients with Diabetes Mellitus: A Systematic Review with Meta-Analysis. BMJ Open Diabetes Res Care.

[73] Zapatero A, Gómez-Huelgas R, González N, Canora J, Asenjo A, Hinojosa J (2014). Frequency of hypoglycemia and its impact on length of stay, mortality, and short-term readmission in patients with diabetes hospitalized in internal medicine wards. Endocr Pract.

[74] Bach QN, Gilmore RA, Sheffield MC, Hawkins WA (2017). Evaluation of insulin use and hypoglycemia in hospitalized elderly patients. Hosp Pharm.

[75] Tosi F, Muggeo M, Brun E, Spiazzi G, Perobelli L, Zanolin ME (2003). Combination treatment with metformin and Glibenclamide versus single-drug therapies in type 2 diabetes mellitus: a randomized, double-blind, comparative study. Metab Clin Exp.

[76] Nachum Z, Zafran N, Salim R, Hissin N, Hasanein J, Gam Ze Letova Y (2017). Glyburide Versus Metformin and Their Combination for the Treatment of Gestational Diabetes Mellitus: A Randomized Controlled Study. Diabetes Care.

[77] Regassa LD, Gete YK, Mekonnen FA (2019). Time to acute kidney injury and its predictors among newly diagnosed type 2 diabetic patients at government hospitals in Harari region, East Ethiopia. PLoS One.

